# Adventitial Delivery of Lentivirus-shRNA-ADAMTS-1 Reduces Venous Stenosis Formation in Arteriovenous Fistula

**DOI:** 10.1371/journal.pone.0094510

**Published:** 2014-04-14

**Authors:** Evelyn C. Nieves Torres, Binxia Yang, Rajiv Janardhanan, Akshaar Brahmbhatt, Ed Leof, Debabrata Mukhopadhyay, Sanjay Misra

**Affiliations:** 1 Vascular and Interventional Radiology Translational Laboratory, Department of Radiology, Mayo Clinic, Rochester, Minnesota, United States of America; 2 Department of Biochemistry and Molecular Biology, Mayo Clinic, Rochester, Minnesota, United States of America; Peking University, China

## Abstract

Hemodialysis vascular access can develop venous neointimal hyperplasia (VNH) causing stenosis. Recent clinical and experimental data has demonstrated that there is increased expression of a disintegrin and metalloproteinase thrombospondin motifs-1 (ADAMTS-1) at site of VNH. The experiments outlined in the present paper were designed to test the hypothesis that targeting of the adventitia of the outflow vein of murine arteriovenous fistula (AVF) using a small hairpin RNA that inhibits ADAMTS-1 expression (LV-shRNA-ADAMTS-1) at the time of fistula creation will decrease VNH. At early time points, ADAMTS-1 expression was significantly decreased associated with a reduction in vascular endothelial growth factor-A (VEGF-A) and matrix metalloproteinase-9 (MMP-9) (LV-shRNA-ADAMTS-1 transduced vessels vs. controls). These changes in gene and protein expression resulted in favorable vascular remodeling with a significant increase in mean lumen vessel area, decrease in media/adventitia area, with a significant increase in TUNEL staining accompanied with a decrease in cellular proliferation accompanied with a reduction in CD68 staining. Collectively, these results demonstrate that ADAMTS-1 transduced vessels of the outflow vein of AVF have positive vascular remodeling.

## Introduction

There are more than 400,000 patients in the United States who require hemodialysis because of end-stage renal disease (ESRD) [1]. A well-functioning vascular access is required for optimal hemodialysis to occur. Current recommendations by National Kidney Foundation Kidney Disease Outcomes Quality Initiative (KDOQI) for hemodialysis vascular access are that patients whom require long-term dialysis have an arteriovenous fistula (AVF). However, it is estimated that at one year patency of AVF's is 62%. [2], [3]. AVFs fail because of venous stenosis formation which is caused by venous neointimal hyperplasia (VNH) [4]. Consequently, treatment of stenosis to maintain the function of hemodialysis AVFs and grafts costs over one billion dollars annually [1]. Developing therapies that could be used to reduce AVF stenosis would be advantageous to ESRD patients.

A recent study from our laboratory demonstrated that there is increased expression of a disintegrin and metalloproteinase with thrombospondin motif-1 (ADAMTS-1) in specimens removed from patients with failed hemodialysis vascular access and experimental animal models of AVF, but its role in the formation of VNH is unknown [5], [6], [7]. A disintegrin and metalloproteinase with thrombospondin motif belongs to a new family of matrix metalloproteinases (MMPs) that were initially described from a colon cancer cell line [8]. ADAMTS-1 has been shown to be able to cleave aggrecan and versican that are components of extracellular matrix. Recent studies have shown that there is increased expression of ADAMTS-1 in plaques from patients with acute myocardial infarction which is associated with increased CD68 staining [9].

The experiments outlined using the present manuscript were performed in a murine model of CKD with AVF to test the hypothesis that reduction of ADAMTS-1 gene expression by adventitial delivery to the outflow vein of the AVF at the time of placement would lead to a reduction in VNH. Gene expression for ADAMTS-1 was reduced by adventitial delivery of a small hairpin RNA (shRNA) that inhibits its expression. Gene, protein expression, and histomorphometric analyses were performed at the outflow vein after administration of anti-ADAMTS-1 RNA therapy.

## Materials and Methods

### Experimental animals

Mayo Clinic Institutional Animal Care and Use Committee approval was obtained prior to performing any procedures. The housing and handling of the animals was performed in accordance with the Public Health Service Policy on Humane Care and Use of Laboratory Animals revised in 2000 [10]. We have previously described this animal model and used it extensively [7], [11], [12], [13]. Briefly, male C57BL/6 mice (Jackson Laboratories, Bar Harbor, ME) weighing 25–30 grams were used for the present study. Chronic renal insufficiency was created as described previously ^20,^ [14]. Four weeks after the nephrectomy, the right carotid artery to the ipsilateral jugular vein was used to create the AVF [11], [14]. Five million particle forming units (PFU) of either lentivirus-shRNA-ADAMTS-1 (**LV-shRNA-ADAMTS-**1) or scrambled-shRNA (**control shRNA**) in 5-μL of PBS were injected using a 30-guage needle, into the adventitia of the proximal outflow vein at the time of AVF creation at the time of fistula creation [15]. We have previously shown that in this animal model that venous stenosis forms reproducibly at this location [7], [11], [12], [13]. Animals were sacrificed at day 7 (**D7**), day 14 (**D14**), and day 21 (**D21**) following AV fistula placement. Real time quantitative polymerase chain reaction (qPCR) and histologic analyses were obtained.

### Vector constructs

The shRNA for ADAMTS-1 and control shRNA were obtained from Open Biosystems, Huntsville, AL (www.openbiosystems.com, RMM4534-NM_001025250) and the lentivirus was prepared according to the manufacturer's protocol. The shRNA for ADAMTS-1 and controls were tagged to GFP (green fluorescent protein) thus allowing for it to be localized after delivery

### Cell culture

We determined the efficacy and efficiency of lentiviral silencing of ADAMTS-1 expression in NIH 3T3 cells which were transduced with either lentivirus-shRNA- ADAMTS-1 (LV-shRNA-VEGF-A) or control shRNA. The gene expression of ADAMTS-1 was determined using RT-PCR [16].

### Tissue harvesting

At euthanasia, all mice were anesthetized as described previously and the fistula was dissected free of the surrounding tissue. Animals were euthanized by CO_2_ asphyxiation and the outflow veins harvested for qPCR or histologic analyses. For histologic analysis, all vessels were perfusion fixed prior to removal.

### Procedures to ensure animal comfort and anesthesia

Mayo strives to ensure that the institutional facilities and procedures adhere in all respects to USDA regulations and NIH guidelines for care and use of laboratory animals. Investigators are required to administer appropriate analgesics to all animals associated with a procedure that would normally require pain medication in humans. The mice undergoing surgery for the creation of the arteriovenous fistula procedure were anesthetized by administering intraperitoneal injection of a mixture of ketamine hydrochloride and xylazine per IACUC recommendations. Surgery was conducted in a disinfected, uncluttered area, which promotes asepsis during surgery. The animals were maintained at a surgical plane of anesthesia throughout the procedure in which the animal is maintained and the vital signs are monitored. The surgical incision was closed using appropriate techniques and materials. After surgery the animal was moved to a warm, dry area and monitored during recovery. Heat lamps or warming pads were used in maintaining or recovering body temperature. All surgical procedures were performed under deep anesthesia with intraperitoneal pentobarbital (20-40 mg/kg). This anesthesia was maintained animal comfort throughout the surgical procedure until recovery or sacrifice.

### RNA isolation

The tissue was stored in RNA stabilizing reagent (Qiagen, Gaithersburg, MD) as per the manufactures guidelines. To isolate the RNA, the specimens were homogenized and total RNA isolated using RNeasy mini kit (Qiagen) as described previously [11], [14].

### Real time polymerase chain reaction (qPCR) analysis

Expression for the gene of interest was determined using qPCR analysis as described previously [11], [17]. Primers used are shown in table 1.

**Table 1:** Q-PCR primers used.

Gene	Sequence
ADAMTS-1	5′ – ACGCCACACTTATCAAACTTCT – 3′ (sense)
	5′ – CTTTTTCGTCTTACAGCCCAAG– 3′ (antisense)
VEGF-A	5′ – ATGAAGTGATCAAGTTCATGG– 3′(sense)
	5′ – GGATCTTGGACAAACAAATGC– 3′ (antisense)
MMP-9	5′ – GTTTTTGATGCTATTGCTGAGATCCA– 3′ (sense)
	5′ – CCCACATTTGACGTCCAGAGAAGAA-3′(antisense)
18S	5′ – GTTCCGACCATAAACGATGCC-3′ (sense)
	5′- TGGTGGTGCCCTTCCGTCAAT- 3′(antisense)

### Tissue processing and immunohistochemistry

Each outflow vein from each animal was embedded in paraffin length-wise so that the sections would be orthogonal to the long axis of the vessel as described previously [17]. Four-μm sections from the outflow vein after transduction with either LV-shRNA-ADAMTS-1 or control shRNA were stained with hematoxylin and eosin, Ki-67, α-SMA, CD31, CD68, MMP-9, ADAMTS-1, and VEGF-A using the EnVision (DAKO, Carpinteria, CA) method with a heat-induced antigen retrieval step [18], [19]. The following antibodies were used: mouse monoclonal antibody Ki-67 (DAKO, 1∶400), CD31 (Abcam, Cambridge, MA; 1∶400), rabbit polyclonal antibody to mouse for VEGF-A, ADAMTS-1, CD-68 (assess for macrophage staining), and MMP-9 (Abcam, 1∶600), or rabbit polyclonal antibody to mouse for MMP-9 (Novus Biologics, 1∶200). IgG antibody staining was performed to serve as controls.

### TUNEL staining [17]

TUNEL staining was performed on paraffin-embedded sections from the outflow vein of LV-shRNA-ADAMTS-1 and control shRNA transduced vessels as specified by the manufacturer (DeadEnd Colorimetric tunnel assay system, G7360, Promega). Negative control is shown where the recombinant terminal deoxynucleotidyl transferase enzyme was omitted.

### Picrosirius red staining [17]

Picrosirius red staining was performed on unstained sections from the outflow vein of LV-shRNA-ADAMTS-1 and control shRNA transduced vessels as described elsewhere [17].

### GFP staining in paraffin tissue

Unstained sections from the outflow vein of LV-shRNA-ADAMTS-1 and control shRNA transduced vessels were deparaffinized in three changes of absolute xylene five minutes each and then with H_2_0 two times each for three minutes. Slides were stained using Vectashield Dapi stain (Vector Labs, Burlingame, CA) and a cover placed and imaged immediately using a confocal microscope (LSM 510 META, Carl Zeiss; Thornwood, NY) at a 10×/0.3 objective.

### Morphometry and image analysis [7], [17], [21]

Morphometric and image analysis was performed as described previously. Briefly, 4-μm paraffin embedded sections that had been immunostained for hematoxylin and eosin stains were viewed using an Axioplan 2 Microscope (Zeiss, Oberkochen, Germany) equipped with a Neo-Fluor × 20/0.50 objective. Images were captured and digitized to a minimum of 3090×3900 pixels using a Axiocam camera (Zeiss) [18], [19]. Only images that covered the entire cross-section from each section of the outflow vein that had been transduced with either LV-shRNA-ADAMTS-1 or control shRNA were acquired and analyzed using KS 400 Image Analysis software (Zeiss). To quantify the lumen vessel area and wall vessel area, we used 3 to 5, 4-um paraffin embedded sections removed from the outflow vein for each animal at each time point. Sections were subsequently viewed with an Axioplan 2 Microscope (Zeiss) equipped with a Neo-Fluor × 20/0.50 objective and digitized to capture at least 1030×1300 pixels and cell density determined along with the vessel wall and luminal vessel areas. The area was measured by tracing the vessel wall using an automated program [19]. Ki-67 (brown), CD68 positive (brown), CD31 positive (brown), α-SMA positive (brown), TUNEL positive (brown), VEGF-A (brown), MMP-9 (brown), and ADAMTS-1 positive (brown) were highlighted, in turn, by selecting the appropriate RGB (red-green-blue) color intensity range and then counted. The color intensity was adjusted for each section to account for decreasing intensity of positive staining over time. This was repeated twice to ensure intra-observer variability was less than 10% [7], [13].

### Statistical methods

Data are expressed as mean ± SEM. Multiple comparisons were performed with two-way ANOVA followed by Student *t*-test with post hoc Bonferroni's correction. Significant difference from control value was indicated by *P<0.05, **P<0.01, ^#^ *P*<0.001, or ^##^ *P*<0.0001. JMP version 9 (SAS Institute Inc., Cary, N.C.) was used for statistical analyses.

## Results

### Surgical outcomes

One hundred and nineteen male C57BL/6 mice weighing 25-30 grams underwent right nephrectomy and left upper pole occlusion surgery as described previously [17].

Seven mice died after nephrectomy, two after AVF fistula placement, and fifteen had significant arterial thickening and inflammation such that a new AV fistula could not be placed. Ninety five mice underwent placement of an AVF to connect the right carotid artery to the ipsilateral jugular vein [17]. Next, either 1×10^6^ PFU of LV-shRNA-ADAMTS-1 (**LV**, n = 48) or control shRNA, scrambled-shRNA, (control, **C**, n = 47) was injected into the adventitia of the outflow vein where the stenosis forms in this model [7], [11]. Animals were sacrificed for gene expression, protein, or histologic analyses at 7 (**D7**), 14 (**D14**), and 21 (**D21**) days after AVF placement.

### Serum BUN and creatinine after nephrectomy

In this model it is observed that there is an increase in creatinine levels post-nephrectomy when compared to non-nephrectomized animals [12], [13].

### Adventitial transduction of LV-shRNA-ADAMTS-1 to the outflow vein reduces gene and protein expression of ADAMTS-1 at 7 and 14 days after AVF placement

The efficacy of reducing ADAMTS-1 gene expression *in vitro* was first determined using NIH 3T3 cells that were transduced with either LV-shRNA-ADAMTS-1 or control shRNA. RT-PCR (**Figure S1**) demonstrated greater than two fold decrease in ADAMTS-1 expression in the LV-shRNA-ADAMTS-1 transduced cells when compared to controls.

**Figure 1:**
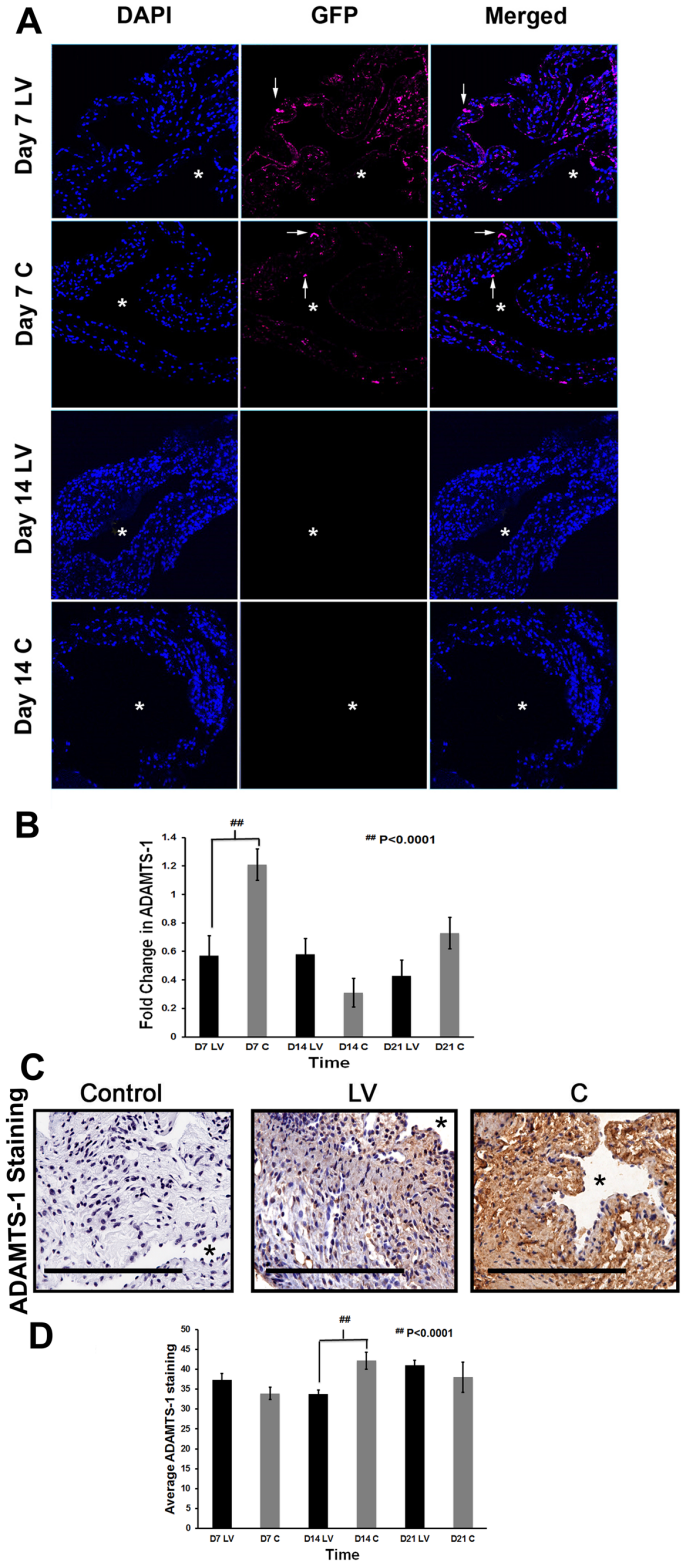
ADAMTS-1 expression is reduced in LV-shRNA-ADAMTS-1 transduced vessels. **A**) is representative sections using confocal microscopy from the outflow vein after transduction with LV-shRNA-ADAMTS-1 (**LV**) and control shRNA (**C**) at day 7 (**D7**) and 14 (**D14**). Both the **C** and **LV** have a GFP tag. First column shows DAPI intensity indicated by blue staining nuclei. Second column shows GRP intensity. Magenta staining cells are GFP positive. Third column depicts merged images. These sections demonstrate that there is **LV** or **C** delivery to the adventitia and endothelium of the vessel wall. By day 14, the gfp signal decreases and is not detectible. **B**) is the pooled data of the mean gene expression of ADAMTS-1 at the outflow vein after transduction with LV-shRNA-ADAMTS-1 (**LV**) compared to control shRNA (**C**) using qPCR analysis at day 7 (**D7**), 14 (**D14**), and 21 (**D21**). This demonstrates a significant reduction in the mean ADAMTS-1 expression in the **LV** transduced vessels when compared to **C** vessels at **D7** (P<0.0001). **C**) is representative sections from ADAMTS-1 staining at the outflow vein of **LV** transduced vessels when compared to **C** vessels at day 14. Cells staining brown are positive for ADAMTS-1. IgG antibody staining was performed to serve as negative control. **D**) is the pooled data of the mean ADAMTS-1 staining at the outflow vein after transduction with **LV** compared to **C** at day 7 (**D7**), 14 (**D14**), and 21 (**D21**). This shows that by **D14**, there is a significant reduction in the mean ADAMTS-1 staining in the **LV** transduced vessels when compared to **C** (P<0.0001). All are 40X. Scale bar is 50-μM. * Indicates the lumen. Each bar shows mean ± SEM of 4-6 animals per group (**B, D**). Two-way ANOVA followed by Student *t*-test with post hoc Bonferroni's correction was performed. Significant difference from control value was indicated by ^##^ *P*<0.0001.

To determine, if similar findings are present *in vivo*, experiments were conducted to determine the distribution of the lentivirus in the outflow vein after delivery to the vessel wall. GFP staining was used to localize the delivery of the lentivirus to the vessel wall. This demonstrated that GFP positive cells (magenta positive) were present at 7 days after adventitial transduction but not at 14 days (**Fig. 1A**). The amount of reduction of ADAMTS-1 gene expression was determined *in vivo* using qPCR analysis for ADAMTS-1 on sections removed from the outflow vein at days 7, 14, and 21 after lentiviral transduction. By day 7, the mean gene expression of ADAMTS-1 at the LV-shRNA-ADAMTS-1 transduced vessels was significantly lower than the control vessels (0.31±0.1 vs. 1.21±0.11, respectively, P<0.0001, Average reduction: 74%, **Fig. 1B**).

It is well known that the expression of the protein can lag behind the decrease in gene expression. Therefore, the protein expression of ADAMTS-1 was determined using immunohistochemistry on outflow vein specimens removed after transduction with either LV-shRNA-ADAMTS-1 and compared to control shRNA (**Fig. 1C**). Cells staining brown are positive for ADAMTS-1. By day 14, there was a significant reduction in the average ADAMTS-1 staining in the LV-shRNA-ADAMTS-1 transduced vessels when compared to controls (33.7±1.06 vs. 42.1±2.2, respectively, P<0.0001, Average reduction: 44%, **Fig. 1D**). By day 21, there was no difference between the two groups.

### Adventitial transduction of LV-shRNA-ADAMTS-1 to the outflow vein promotes positive vascular remodeling with a decrease in constrictive remodeling

**Figure 2:**
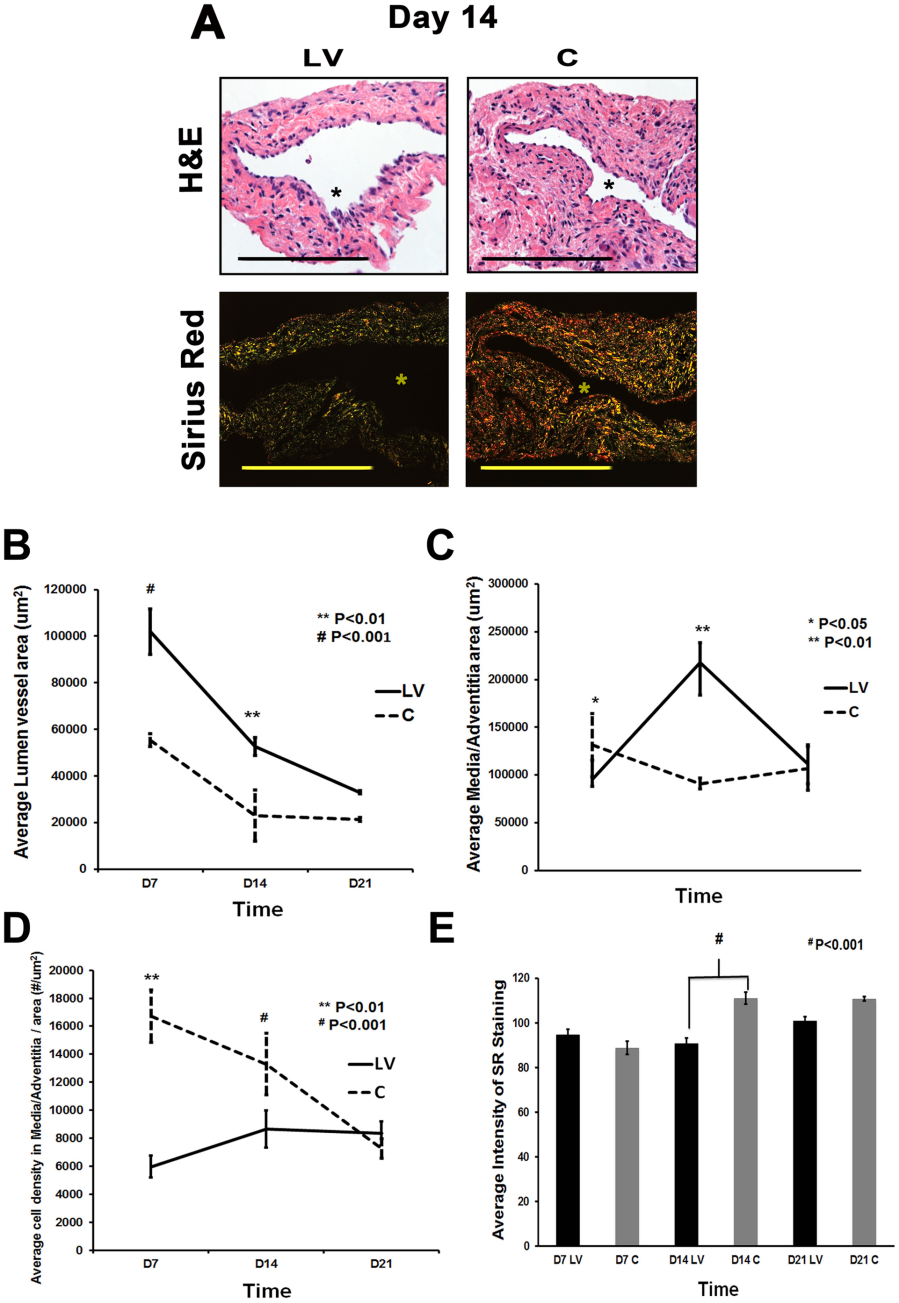
Hematoxylin and eosin (H&E) and picrosirius red staining of LV-shRNA-ADAMTS-1 transduced vessels have increased lumen vessel area with decreased collagen expression. **A) Upper panel** is representative sections after hematoxylin and eosin (H&E) at the venous stenosis of the LV-shRNA-ADAMTS-1 (**LV**) and control shRNA (**C**) transduced vessels at day at day 14 showing increase in lumen vessel area. **A**) **Lower panel** is representative polarized light microscopy of picrosirius red-stained venous stenosis showing decreased fibrosis (collagen fibers are bright yellow) of the **LV** and **C** transduced vessels. Pooled data for the average lumen vessel area of the **LV** and **C** groups at day 7 (**D7**), 14 (**D14**), and 21 (**D21**) are shown in **B**). There is a significant increase in the average lumen vessel area in the **LV** transduced vessels when compared to **C** vessels for day 7 (P<0.001) and day 14 (P<0.01). Pooled data for average area of the media and adventitia for **LV** and **C** groups at day 7 (**D7**), 14 (**D14**), and 21 (**D21**) are shown in (**C**). By day 7, there is a significant increase in the average area of the media and adventitia in the **LV** transduced vessels when compared to **C** vessels (P<0.05). By day 14, the average area of the media and adventitia is significantly increased in the **LV** transduced vessels when compared to **C** vessels (P<0.01). Pooled data for the average cell density in the media and adventitia in the **LV** and **C** groups at day 7 (**D7**), 14 (**D14**), and 21 (**D21**) are shown in (**D**). There is a significant decrease in average cell density in the media and adventitia in the **LV** transduced vessels when compared to **C** vessels for day 7 (P<0.01) and day 14 (P<0.001). Pooled data for average intensity of picrosirius red staining of the vessel wall for **LV** and **C** groups at day 7 (**D7**), 14 (**D14**), and 21 (**D21**) are shown in (**E**). There is a significant decrease in average intensity of picrosirius red staining of the vessel wall of **LV** transduced vessels when compared to **C** vessels for day 14 (P<0.001). All are 40X. Scale bar is 50-μM. * Indicates the lumen. Each bar shows mean ± SEM of 4-6 animals per group (**B-E**). Two-way ANOVA followed by Student *t*-test with post hoc Bonferroni's correction was performed. ^*^ *P*<0.05, ^**^ *P*<0.01, or ^#^ *P*<0.001.

**Figure 3:**
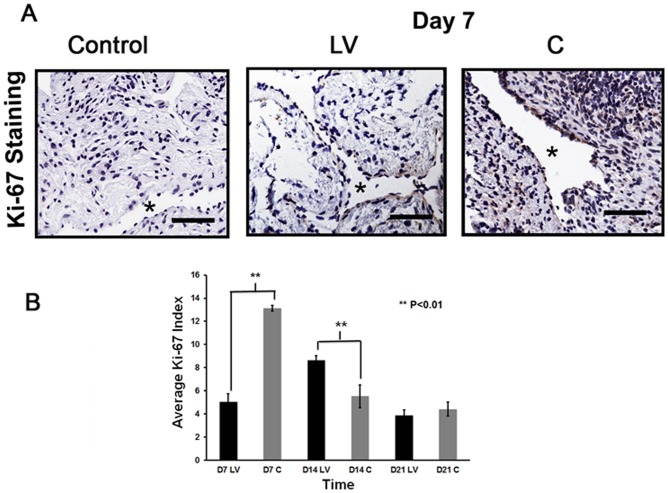
Cellular proliferation is decreased in LV-shRNA-ADAMTS-1 transduced vessels. **A**) is representative sections after Ki-67 staining at the venous stenosis of the LV-shRNA-ADAMTS-1 (**LV**) and control shRNA (**C**) at day 7. Nuclei staining brown are positive for Ki-67. IgG antibody staining was performed to serve as negative control. All are 40X. Scale bar is 50-μM. * indicates the lumen. Pooled data for Ki-67 index at the outflow vein of the **LV** and **C** transduced vessels at day 7 (**D7**), 14 (**D14**), and 21 (**D21**) is shown in (**B**). By day 7, there is a significant decrease in the mean Ki-67 index in the **LV** transduced vessels when compared to **C** at day 7 (P<0.01). By day 14, the mean Ki-67 index in the **LV** transduced vessels is significantly increased when compared to **C** (P<0.01). Each bar shows mean ± SEM of 4-6 animals per group. Two-way ANOVA followed by Student *t*-test with post hoc Bonferroni's correction was performed. Significant difference from control value was indicated by *P<0.01.

We hypothesized that reducing mRNA levels of ADAMTS-1 by adventitial delivery of LV-shRNA-ADAMTS-1 to the outflow vein would result in positive vascular remodeling at the outflow vein (**Fig. 2** **)**. Hematoxylin and eosin staining (H&E) (**Fig. 2A** **upper row**) and picrosirius red staining (**Fig. 2A** **lower row**) was used to assess the histomorphometric changes in vascular remodeling. A representative H&E and picrosirius red stains are shown from day 14. There was a significant increase in the average lumen area of the outflow vein of the LV-shRNA-ADAMTS-1 transduced vessels when compared to the control vessels by day 7 (average increase: 184%, P<0.001) and day 14 (average increase: 229%, P<0.01, **Fig. 3B**). Adventitial remodeling has been observed in experimental models of VNH associated with hemodialysis vascular access [18], [20], [21]. This was assessed by measuring the area of the adventitia and media. By day 7, there was a significant reduction in the average area of the media and adventitia of the LV-shRNA-ADAMTS-1 transduced vessels when compared to controls (average decrease: 27%, P<0.05, **Fig. 2C**). By day 14, there was a significant increase in the average area of the media and adventitia (average increase: 139%, P<0.01) with no differences in the average neointima area between the two groups.

A decrease in cell density can accompany positive vascular remodeling [12], [13]. By day 7, there was a significant reduction in the average cell density of the media and adventitia of the LV-shRNA-ADAMTS-1 transduced vessels when compared to controls (average decrease: 55%, P<0.05, **Fig. 2D**). By day 14, there was a significant increase in the average cell density in the LV-shRNA-ADAMTS-1 transduced vessels when compared to controls (average increase: 238%, P<0.01).

Picrosirius staining was used to assess changes in collagen types 1 and 3 content in the LV-shRNA-ADAMTS-1 transduced vessels when compared to controls. Yellow color is positive for collagen types 1 or 3 staining. By day 14, there was a significant decrease in the average picrosirius staining in the LV-shRNA-ADAMTS-1 transduced vessels when compared to controls (Average reduction: 18%, P<0.001, **Fig. 2E**).

### Adventitial transduction of LV-shRNA-ADAMTS-1 to the outflow vein decreases cellular proliferation

Cellular proliferation was determined by performing Ki-67 staining on sections from the outflow vein after transduction with either LV-shRNA-ADAMTS-1 or control shRNA (**Fig. 3A**). By day 7, the average Ki-67 index in the LV-shRNA-ADAMTS-1 group was significantly lower than the control group (5.1±0.7 vs. 9.3±1.7, respectively, average reduction: 45%, P<0.01, **Fig. 3B**). By day 14, a significant increase in the average Ki-67 index in the LV-shRNA-ADAMTS-1 transduced group was observed when compared to control group (8.65±0.4 vs. 5.5±0.98, respectively, average increase: 158%, P<0.01).

### Adventitial transduction of LV-shRNA-ADAMTS-1 to the outflow vein increases apoptosis at day 14 and 21

**Figure 4:**
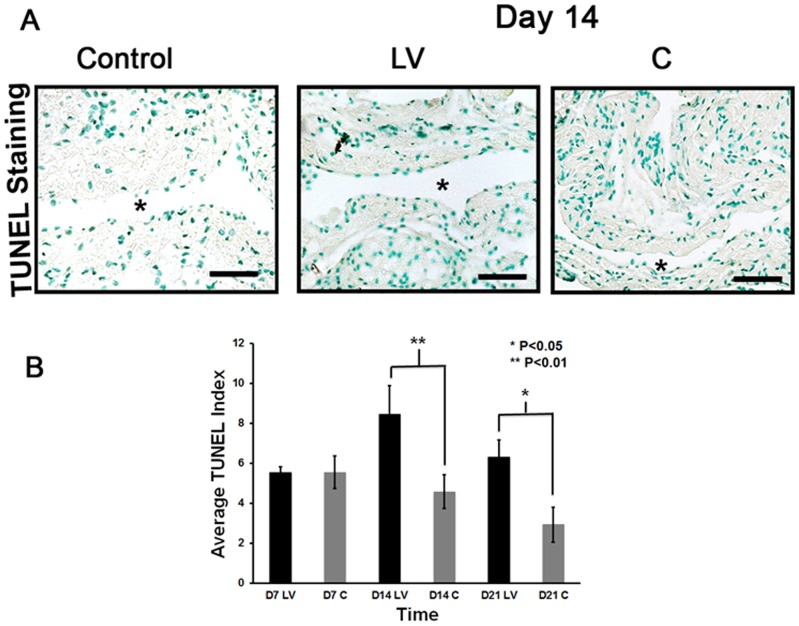
Apoptosis is increased in the LV-shRNA-ADAMTS-1 transduced vessels. **A**) is a representative section from TUNEL staining at the venous stenosis of the LV-shRNA-ADAMTS-1 (**LV**) and control shRNA(**C**) transduced control vessels at day 14. Nuclei staining brown are positive for TUNEL. Negative control is shown where the recombinant terminal deoxynucleotidyl transferase enzyme was omitted. All are 40X. Scale bar is 50-μM. * Indicates the lumen. Arrow indicates TUNEL positive cells. Pooled data for **LV** and **C** transduced vessels for TUNEL index at the outflow vein of the **LV** and **C** transduced vessels at day 7 (**D7**), 14 (**D14**), and 21 (**D21**) is shown in **(B)**. This demonstrates a significant increase in the mean TUNEL index at day 14 (P<0.01) and day 21 (P<0.05) in the **LV** group when compared to **C**. Each bar shows mean ± SEM of 3–6 animals per group. Two-way ANOVA followed by Student *t*-test with post hoc Bonferroni's correction was performed. Significant difference from control value was indicated by *P<0.05 or **P<0.01.

Apoptosis was assessed using TUNEL staining performed on sections removed after transduction with either LV-shRNA-ADAMTS-1 or control shRNA (**Fig. 4A**). The average TUNEL index (number of TUNEL positive cells (brown)/total number of cells ×100) at the outflow vein of the LV-shRNA-ADAMTS-1 group was significantly higher than the control group by day 14 (8.4±1.4 vs. 4.6±0.8, respectively, average increase: 185%, P<0.01, **Fig. 4B**) and day 21 (6.3±0.9 vs. 2.9±0.9, respectively, average increase: 215%, P<0.05). Overall, these results indicate that adventitial delivery of LV-shRNA-ADAMTS-1 results in a significant increase in TUNEL activity suggesting that there is increased cell death in LV-shRNA-ADAMTS-1 transduced vessels when compared to controls.

### Adventitial transduction of LV-shRNA-ADAMTS-1 to the outflow vein decreases CD68 staining at day 7

**Figure 5:**
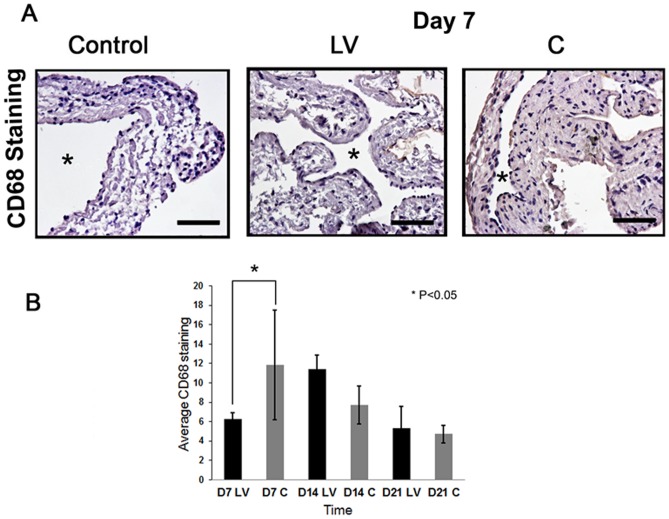
CD68 expression is reduced in LV-shRNA-ADAMTS-1 transduced vessels. **A)** is a representative section from CD68 staining at the venous stenosis of the LV-shRNA-ADAMTS-1 (**LV**) and control shRNA (**C**) transduced control vessels at day 7. Cells staining brown are positive for CD68. IgG antibody staining was performed to serve as negative control. All are 40X. Scale bar is 50-mM. * Indicates the lumen. Arrow indicates TUNEL positive cells. Pooled data for **LV** and **C** transduced vessels for CD68 index at the outflow vein of the **LV** and **C** transduced vessels at day 7 (**D7**), 14 (**D14**), and 21 (**D21**) is shown in **(B)**. This demonstrates a significant increase in the mean TUNEL index at day 74 (P<0.05) in the **LV** group when compared to **C**. Each bar shows mean ± SEM of 3-6 animals per group. Two-way ANOVA followed by Student *t*-test with post hoc Bonferroni's correction was performed. Significant difference from control value was indicated by *P<0.05.

Studies have demonstrated that ADAMTS-1 expression is associated with CD68 staining [9], [22]. CD68 staining was performed to assess the changes after LV-shRNA-ADAMTS-1 transduction at different time points (**Fig. 5A**). This demonstrated that there was a significant decrease in the average CD68 staining at day 7 in the LV-shRNA-ADAMTS-1 transduced vessels when compared to controls (6.29±0.60 vs. 11.9±5.6, respectively, average reduction: 47%, P<0.05, **Fig. 5B**). By day 14, the CD68 staining had increased in the LV-shRNA-ADAMTS-1 transduced vessels when compared to controls however it was not statistically significant.

### Adventitial delivery of LV-shRNA-ADAMTS-1 to the outflow vein is associated with a reduction of gene and protein expression of VEGF-A at days 7 and 14

**Figure 6:**
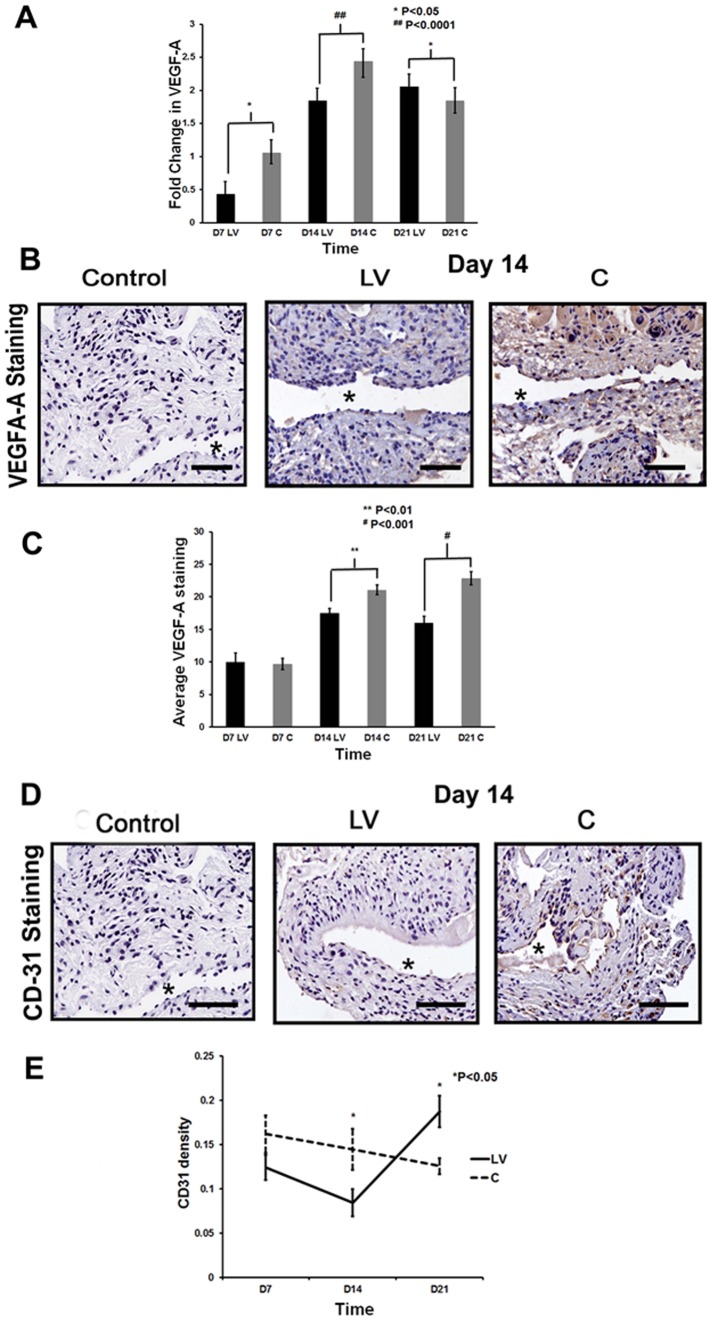
VEGF-A expression is reduced in LV-shRNA-ADAMTS-1 transduced vessels. **A**) is the pooled data from the mean gene expression of VEGF-A at the outflow vein after transduction with LV-shRNA-ADAMTS-1 (**LV**) compared to control shRNA (**C**) using qPCR analysis at day 7 (**D7**), 14 (**D14**), and 21 (**D21**). This demonstrates that there is significant reduction in the mean VEGF-A expression in the **LV** transduced vessels when compared to **C** vessels at day 7 (P<0.01) and day 14 (P<0.001). By day 21, there is a significant increase in VEGF-A gene expression in the **LV** treated vessels when compared to **C** vessels (P<0.0.001). **B**) is representative sections from VEGF-A staining at the venous stenosis of the **LV** and **C** transduced vessels at day at day 14. Cells staining brown are positive for VEGF-A. IgG antibody staining serves as negative control. **C**) shows that there is a significant reduction in the mean VEGF-A staining in the **LV** transduced vessels when compared to controls by day 14 (P<0.01) and 21 (P<0.001). **D**) are representative sections from CD-31 staining at the venous stenosis of the LV-shRNA-ADAMTS-1 (**LV**) and control shRNA (**C**) transduced vessels at day at day 14. Cells staining brown are positive for CD31. IgG antibody staining was performed to serve as negative control. **E**) shows that by day 14, there is a significant reduction in the mean CD31 staining in the **LV** transduced vessels when compared to controls by day 14 (P<0.05). By day 21, there is a significant increase in the mean CD31 staining in the **LV** transduced vessels when compared to **C** (P<0.05). All are 40X. Scale bar is 50-μM. * Indicates the lumen. Each bar shows mean ± SEM of 4–6 animals per group (**A, C**, and **E**). Two-way ANOVA followed by Student *t*-test with post hoc Bonferroni's correction was performed. Significant difference from control value was indicated by ^*^ *P*<0.05 or ^#^ *P*<0.001.

There have been studies that have shown that ADAMTS-1 can modulate the VEGF-A response. This was assessed using qPCR analysis for VEGF-A expression at the outflow vein at days 7, 14, and 21 after lentiviral transduction [23]. The mean gene expression of VEGF-A at the LV-shRNA-ADAMTS-1 transduced vessels was significantly lower than the control vessels by day 7 (0.29±0.17 vs. 1.06±0.17, respectively, P<0.05, Average reduction: 73%, **Fig. 6A**) and 14 (0.43±0.21 vs. 2.44±0.17, respectively, P<0.0001, Average reduction: 83%). By day 21, the mean VEGF-A expression increased significantly in the LV-shRNA-ADAMTS-1 transduced vessels when compared to controls (1.84±0.17 vs. 1.19±0.17, respectively, P<0.05, Average increase: 154%).

To determine the consequence of the reduction in gene expression of VEGF-A, protein expression of VEGF-A was assessed using immunohistochemistry after lentiviral mediated gene silencing of ADAMTS-1 (**Fig. 6B**). Cells staining brown are positive for VEGF-A. There was a significant reduction in the average VEGF-A staining at the LV-shRNA-ADAMTS-1 transduced vessels when compared to controls by day 14 (17.5±0.7 vs. 21±0.7, respectively, P<0.01, Average reduction: 17%, **Fig. 6C**) and 21 (16±0.97 vs. 22.8±1.02, respectively, P<0.001, Average reduction: 30%).

CD31 staining can be used to assess the formation of new blood vessel formation after anti-angiogenic therapy [12]. Cells staining brown are positive for CD31. The effect of VEGF-A reduction on angiogenesis in LV-shRNA-ADAMTS-1 transduced vessels or control shRNA vessels were assessed using CD31 staining (**Fig. 6D**). By day 14, there was a significant decrease in the average CD31 density in the LV-shRNA-ADAMTS-1 transduced vessels when compared to controls (0.08±0.02 vs. 0.14±0.02, respectively, P<0.05, Average reduction: 42%). By day 21, there was a reversal of the average CD31 density with an increase in the LV-shRNA-ADAMTS-1 transduced vessels when compared to controls (0.19±0.02 vs. 0.13±0.01, respectively, P<0.05, Average increase: 149%, **Fig. 6E**). Collectively, these results indicate that at early time points, ADAMTS-1 silencing is associated with a reduction in the mean gene expression and subsequent protein expression of VEGF-A which is accompanied with a decrease in CD31 density. At later time points, there is an increase in the mean gene expression of VEGF-A expression and CD31 density.

### Adventitial transduction of LV-shRNA-ADAMTS-1 to the outflow vein reduces expression of MMP-9 at the outflow vein

**Figure 7:**
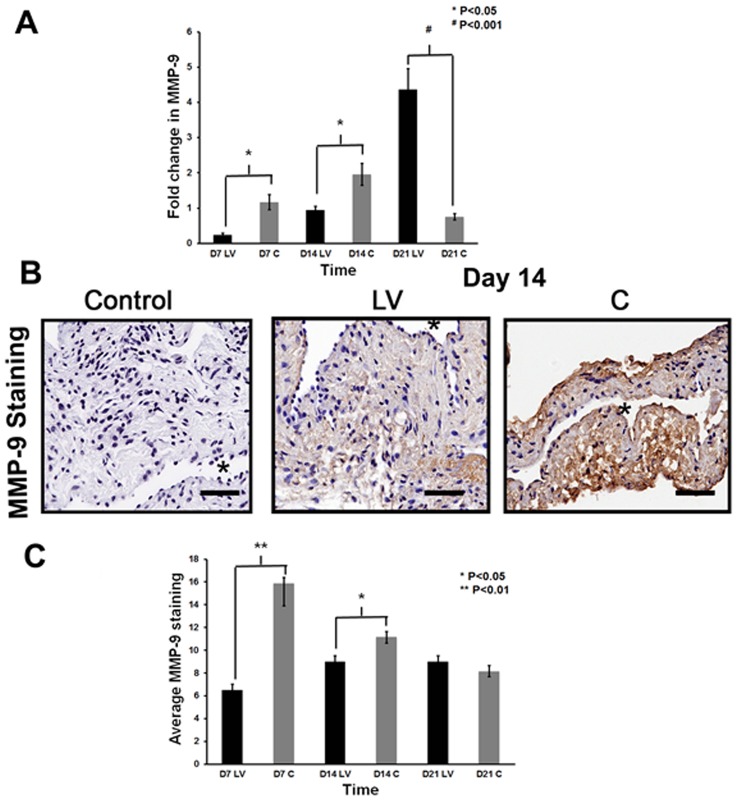
MMP-9 expression is decreased in LV-shRNA-ADAMTS-1 transduced vessels. **A**) is pooled data for qPCR analysis for MMP-9 expression of the LV-shRNA-ADAMTS-1 (**LV**) and control shRNA (**C**) transduced vessels at day 7 (**D7**), 14 (**D14**), and 21 (**D21**). This demonstrates a significant reduction in the average expression of MMP-9 in the **LV** transduced vessels when compared to **C** at day 7 (P<0.05) and 14 (P<0.05) with a significant increase in MMP-2 by day 28 (P<0.001). **B**) are representative sections from MMP-9 staining at the venous stenosis of the **LV** and **C** transduced vessels at day at day 14. Cells staining brown are positive for MMP-9. IgG antibody staining was performed to serve as negative control. **C**) is pooled data for the average MMP-9 expression at the outflow vein of the **LV** and **C** transduced vessels at day 7 (**D7**), 14 (**D14**), and 21 (**D21**). This shows that there is a significant reduction in the mean MMP-9 staining in the **LV** transduced vessels when compared to controls by day 7 (P<0.01) and day 14 (P<0.05). All are 40X. Scale bar is 50-μM. * Indicates the lumen. Each bar shows mean ± SEM of 3-6 animals per group (**A, C**). Two-way ANOVA followed by Student *t*-test with post hoc Bonferroni's correction was performed. Significant difference from control value was indicated by *P<0.05 or ^#^ *P*<0.001.

Recent study has shown that a decrease in VEGF-A is associated with a decrease in MMP-9 [12]. In addition, several studies have shown that there is increased expression of MMP-9 in animal models of hemodialysis AVF and graft failure as well as clinical samples [14], [18], [19], [24], [25]. We determined the gene expression of MMP-9 using qPCR analysis on specimens removed from the outflow vein transduced with either LV-shRNA-ADAMTS-1 or control shRNA. The average gene expression of MMP-9 was significantly lower in the LV-shRNA-ADAMTS-1 transduced vessels when compared to control shRNA by day 7 (0.24±0.06 vs. 1.17±0.21, respectively, average reduction: 80%, P<0.05, **Fig. 7A**) and 14 (0.94±0.11 vs. 1.96±0.31, respectively, average reduction: 52%, P<0.05). By day 21, there was a significant increase in MMP-9 expression in LV-shRNA-ADAMTS-1 transduced vessels when compared to controls (4.4±0.6 vs. 0.8±0.09, respectively, average increase: 477%, P<0.001).

The protein expression of MMP-9 was determined using immunohistochemistry performed on the outflow vein transduced with either LV-shRNA-ADAMTS-1 or control shRNA (**Fig. 7B**). The average MMP-9 staining (brown staining cells) was significantly lower in the LV-shRNA-ADAMTS-1 transduced vessels when compared to control vessels (6.52±1.4 vs. 15.9±1.9, respectively, average reduction: 59%, P<0.01, **Fig. 7C**) and by day 14 it remained lower (9±.2 vs. 11.1±.53, respectively, average reduction: 19%, P<0.05).

## Discussion

**Figure 8:**
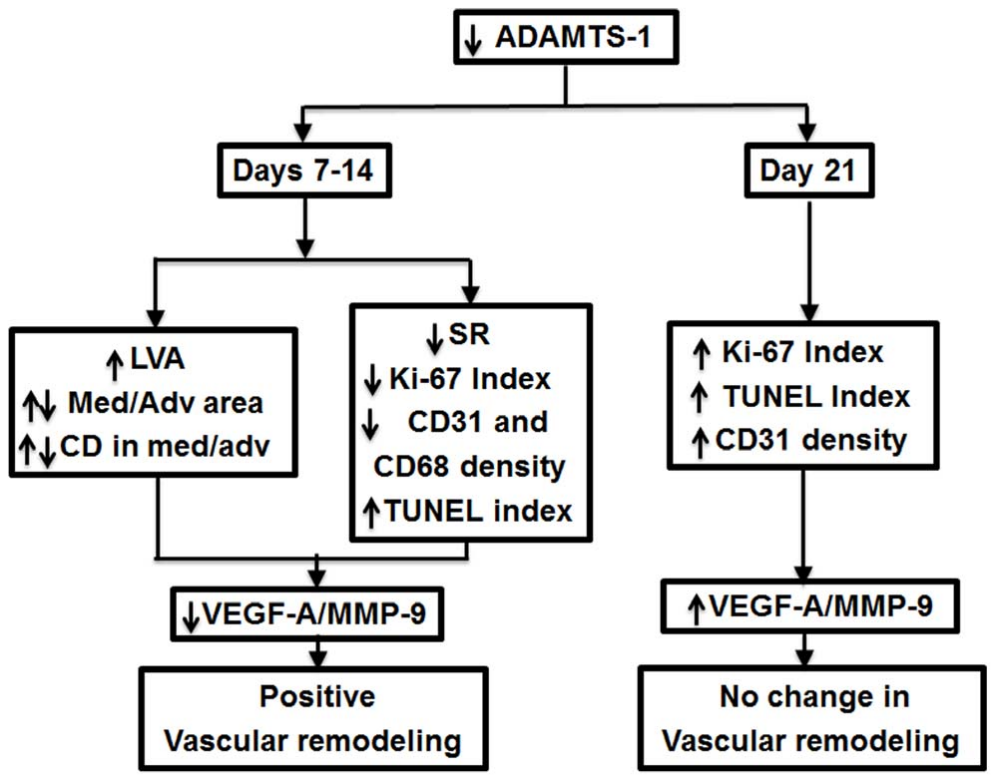
Synopsis of major findings.

Using a proteomic analysis, our laboratory identified ADAMTS-1 as a prominent up-regulated protein in specimens removed from failed hemodialysis grafts, however its role in the formation of VNH remained unknown [6]. In a murine model of AVF with CKD, a significant increase in ADAMTS-1 expression was demonstrated at the outflow vein when compared to controls [7]. In the present study, ADAMTS-1 expression was reduced using a LV-shRNA-ADAMTS-1 administered to the adventitia of the outflow vein at the time of AVF placement. In LV-shRNA-ADAMTS-1 transduced vessels compared to controls, there was a reduction of mRNA for ADAMTS-1 associated with a reduction in mRNA expression of VEGF-A and MMP-9 at days 7 to 14 after adventitial delivery. At day 21, there was a reduction in the protein expression of VEGF-A and MMP-9. Associated with this decrease, the following important histomorphometric changes were observed: 1) significant increase in mean lumen vessel area; 2) significant decrease in average area of the media and adventitia with corresponding cell density; 3) significant decrease in constrictive remodeling; 4) significant decrease in average cellular proliferation; 5) significant increase in TUNEL; and 6) significant decrease in macrophage density. These results suggest that ADAMTS-1 mRNA reduction results in favorable vascular remodeling that is associated with a reduction in VEGF-A (**Fig. 8**).

A disintegrin and metalloproteinase with thrombospondin motif-1 belongs to a new family of matrix metalloproteinases (MMPs) that were initially described from a murine colon cancer cell line [8]. The role of ADAMTS-1 in angiogenesis is controversial [26]. This effect has been demonstrated to occur through the direct binding of ADAMTS-1 to the trans-membrane receptor and thereby inhibiting VEGF-A binding [23], [27]. Other studies have demonstrated the opposite effect [28]. These differences in its actions have led some authors to postulate that there may be a feedback inhibition of angiogenesis by ADAMTS-1 on VEGF-A [29]. In fact, a recent study demonstrated that ADAMTS-1 and its fragments have both pro-angiogenic and anti-angiogenic activities [30]. In the present study, reducing mRNA levels of ADAMTS-1 was associated with a decrease in VEGF-A at early time points while at later time points, the levels of VEGF-A were increased. The reduction of VEGF-A was accompanied with a reduction in MMP-9. Previous work from our laboratory and others has shown that decreasing VEGF-A results in a decrease in MMP-9 [12], [13].

Since VEGF-A is responsible for angiogenesis, we used CD31, a marker for new blood vessel formation, and found the average CD31 to be decreased at early time points in ADAMTS-1 transduced vessels when compared to controls. These observations are consistent with recent work from our laboratory in which reducing VEGF-A mRNA levels has been shown to decrease CD31 expression [12]. Some investigators have hypothesized that there is a link between microvessel formation as depicted by CD31 staining and stenosis formation [31].

The role of ADAMTS-1 in proliferation is also controversial since there are conflicting reports. Some studies have demonstrated that ADAMTS-1 is associated with increased proliferation while others have shown the opposite [27], [29]. In the present study, proliferation was assessed in the vessel wall using Ki-67 staining and found to be decreased in LV-shRNA-ADAMTS-1 transduced vessels when compared to controls. These results are consistent with observations from experimental animal model of vascular injury caused by atherosclerosis involving the carotid artery. In this study, the authors observed a significant increase in mRNA levels of ADAMTS-1 with increase in proliferation and migration [5]. In the present study, the reduction in not only ADAMTS-1 but also VEGF-A and MMP-9 could be responsible for the decrease in proliferation at day 7. Previous studies from our laboratory demonstrated that reducing mRNA for VEGF-A at the outflow vein of AVF decreased cellular proliferation [12]. Other studies have shown that inhibiting MMP activity in a rat and porcine model of arteriovenous hemodialysis graft failure results in a reduction in stenosis formation [32], [33]

Accompanied with a decrease in proliferation, an increase in TUNEL staining was observed. In tumor cell lines, ADAMTS-1 over expression has been shown to have no effect on apoptosis [34]. The role of ADAMTS-1 in cell death in vascular injury has not been investigated. TUNEL staining was used to assess changes of cell death in the vessel wall after LV-shRNA-ADAMTS-1 transduction when compared to controls. The increase in TUNEL staining may represent the decrease in VEGF-A expression. It is well known that VEGF-A is needed for cell survival and that reducing VEGF-A expression results in apoptosis and cell death [12], [35], [36]. Recently, using the same animal model, we observed that reducing VEGF-A mRNA expression using shRNA or statin therapy resulted in an increase in TUNEL staining with increased caspase 3 activity [12], [13].

The role of ADAMTS-1 reduction in vascular remodeling with respect to collagen 1 and 3 changes was assessed using picrosirius red staining. This stain is used to assess collagen types 1 and 3. A decrease in collagen types 1 and 3 implies that there is a reduction in constrictive remodeling. There have been no previous studies, which have demonstrated a link between ADAMTS-1 and collagens 1 and 3. The decrease in collagen 1 and 3 staining was observed in the present study in the LV-shRNA-ADAMTS-1 transduced vessels when compared to controls and we speculate that these changes may reflect the reduction in VEGF-A and other cytokines which were not measured.

A recent demonstrated that there is increased expression of ADAMTS-1 which is associated with CD68 staining in patients with acute myocardial infarction [9]. In the present study, we observed a significant decrease in CD68 staining for macrophages at day 7. However, we did not observe a difference in smooth muscle staining at any of the time points. These results imply that ADAMTS-1 reduction results in macrophage density possibly by reducing macrophage infiltration which has been observed in experimental vascular injury [22].

In conclusion, the present study demonstrates that reducing ADAMTS-1 mRNA expression levels at the time of placement of AVF results in a positive vascular associated with a reduction in macrophage staining. There is a decrease in VEGF-A expression as well as a decrease in MMP-9 expression accompanied with a reduction in proliferation, constrictive remodeling, CD31 staining, and an increase in TUNEL. These findings have implications for the development of translational therapies aimed at inhibiting VNH in patients with AVF and ESRD.

## Significance

There are more than 400,000 patients in the United States who require hemodialysis because of end-stage renal disease (ESRD). Hemodialysis vascular access can develop venous neointimal hyperplasia (VNH) causing access failure. Recent clinical and experimental data has demonstrated that there is increased expression of a disintegrin and metalloproteinase thrombospondin motifs–1 (ADAMTS-1) at site of VNH. Using a murine model of AVF with chronic kidney disease, we reduced ADAMTS-1 mRNA expression with a small hairpin RNA that inhibits ADAMTS-1 expression (LV-shRNA-ADAMTS-1) at the time of fistula creation. This resulted in a decrease in VNH accompanied with a decrease in macrophage staining with no change in smooth muscle density. At early time points, ADAMTS-1 expression was significantly decreased accompanied with a decrease in vascular endothelial growth factor-A (VEGF-A) and matrix metalloproteinase-9 (MMP-9). These changes in gene expression resulted in decrease in media/adventitia area, with a significant increase in TUNEL staining accompanied with a decrease in cellular proliferation.

## Supporting Information

Figure S1 **LV-shRNA-ADAMTS-1 transfection decreases ADAMTS-1 expression in cells.** Gene expression of ADAMTS-1 in NIH3T3 cells transfected with control ShRNA, LV-shRNA-ADAMTS-1, positive control for ADAMTS-1 (**+C**), and a negative control (**-C**) showing greater than two fold decrease in ADAMTS-1 expression with LV-shRNA-ADAMTS-1 silencing when compared to controls.
(TIF)
