# Correction: Adventitial Delivery of Lentivirus-shRNA-ADAMTS-1 Reduces Venous Stenosis Formation in Arteriovenous Fistula

**DOI:** 10.1371/journal.pone.0113312

**Published:** 2014-11-05

**Authors:** The PLOS ONE Staff

Dr. Bhaskar Roy should be included in the author byline. He should be listed as the third author, and his affiliation is 1: Vascular and Interventional Radiology Translational Laboratory, Department of Radiology, Mayo Clinic, Rochester, Minnesota, United States of America. The contributions of this author are as follows: Performed the experiments, analyzed the data.

The correct citation is:

Nieves Torres EC, Yang B, Roy B, Janardhanan R, Brahmbhatt A, et al. (2014) Adventitial Delivery of Lentivirus-shRNA-ADAMTS-1 Reduces Venous Stenosis Formation in Arteriovenous Fistula. PLoS ONE 9(4): e94510. doi:10.1371/journal.pone.0094510
