# Supplementary figures and images for: Adventitial Delivery of Lentivirus-shRNA-ADAMTS-1 Reduces Venous Stenosis Formation in Arteriovenous Fistula
Source: PLoS One. 2014 Apr 14;9(4):e94510. doi: 10.1371/journal.pone.0094510 (PMC3986087; doi:10.1371/journal.pone.0094510)

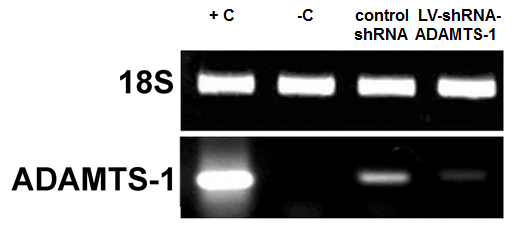

Supplement: Figure S1 — LV-shRNA-ADAMTS-1 transfection decreases ADAMTS-1 expression in cells. Gene expression of ADAMTS-1 in NIH3T3 cells transfected with control ShRNA, LV-shRNA-ADAMTS-1, positive control for ADAMTS-1 (+C), and a negative control (-C) showing greater than two fold decrease in ADAMTS-1 expression with LV-shRNA-ADAMTS-1 silencing when compared to controls. (TIF) [file pone.0094510.s001.tif]
